# Adapting medical guidelines to be patient-centered using a patient-driven process for individuals with sickle cell disease and their caregivers

**DOI:** 10.1186/s12878-018-0106-3

**Published:** 2018-06-08

**Authors:** Robert Michael Cronin, Tilicia L. Mayo-Gamble, Sarah-Jo Stimpson, Sherif M. Badawy, Lori E. Crosby, Jeannie Byrd, Emmanuel J. Volanakis, Adetola A. Kassim, Jean L. Raphael, Velma M. Murry, Michael R. DeBaun

**Affiliations:** 1 Department of Biomedical Informatics, Vanderbilt University Medical Center, 2525 West End Blvd., Suite 1475, Nashville, TN 37232 USA; 2 Department of Internal Medicine, Vanderbilt University Medical Center, Nashville, TN USA; 3 Department of Pediatrics, Vanderbilt University Medical Center, Nashville, TN USA; 4 Department of Family and Community Medicine, Meharry Medical College, Nashville, TN USA; 5 Department of Pediatrics, Division of Hematology/Oncology, Vanderbilt-Meharry Center for Excellence in Sickle Cell Disease, Vanderbilt University Medical Center, Nashville, TN USA; 6 Department of Pediatrics, Division of Hematology, Oncology and Stem Cell Transplant, Ann & Robert H. Lurie Children’s Hospital of Chicago, Northwestern University Feinberg School of Medicine, Chicago, IL USA; 7 Division of Behavioral Medicine, Department of Pediatrics, Cincinnati Children’s Hospital Medical Center and the University of Cincinnati, College of Medicine, Cincinnati, OH USA; 8 Department of Medicine, Division of Hematology/Oncology, Vanderbilt-Meharry Center for Excellence in Sickle Cell Disease, Vanderbilt University Medical Center, Nashville, TN USA; 9 Department of Pediatrics, Section of Hematology-Oncology and Section of Academic General Pediatrics, Baylor College of Medicine, Houston, TX USA; 10 Department of Human & Organizational Development, Vanderbilt University, Nashville, TN USA

**Keywords:** Sickle cell disease, Clinical practice guidelines, Patient-centered, Community-engaged research, Technology, Patient decision making, Qualitative methods

## Abstract

**Background:**

Evidence-based guidelines for sickle cell disease (SCD) health maintenance and management have been developed for primary health care providers, but not for individuals with SCD. To improve the quality of care delivered to individuals with SCD and their caregivers, the main purposes of this study were to: (1) understand the desire for patient-centered guidelines among the SCD community; and (2) adapt guideline material to be patient-centered using community-engagement strategies involving health care providers, community -based organizations, and individuals with the disease.

**Methods:**

From May–December 2016, a volunteer sample of 107 individuals with SCD and their caregivers gave feedback at community forums (*n* = 64) and community listening sessions (*n* = 43) about technology use for health information and desire for SCD-related guidelines. A team of community research partners consisting of community stakeholders, individuals living with SCD, and providers and researchers (experts) in SCD at nine institutions adapted guidelines to be patient-centered based on the following criteria: (1) understandable, (2) actionable, and (3) useful.

**Results:**

In community forums (n = 64), almost all participants (91%) wanted direct access to the content of the guidelines. Participants wanted guidelines in more than one format including paper (73%) and mobile devices (79%). Guidelines were adapted to be patient-centered. After multiple iterations of feedback, 100% of participants said the guidelines were understandable, most (88%) said they were actionable, and everyone (100%) would use these adapted guidelines to discuss their medical care with their health care providers.

**Conclusions:**

Individuals with SCD and their caregivers want access to guidelines through multiple channels, including technology. Guidelines written for health care providers can be adapted to be patient-centered using Community-engaged research involving providers and patients. These patient-centered guidelines provide a framework for patients to discuss their medical care with their health care providers.

**Electronic supplementary material:**

The online version of this article (10.1186/s12878-018-0106-3) contains supplementary material, which is available to authorized users.

## Background

Sickle cell disease (SCD) is an inherited disorder of hemoglobin affecting over 100,000 Americans, many of whom are poor and minorities [1–4]. SCD causes severe complications and has a substantial impact on both the population of affected individuals and the utilization of health care services in the United States; adults with SCD average 197,000 emergency room visits per year, and the lifetime costs of care for the average sickle cell patient are estimated at $900,000 by the age of 45 [4–6]. The primary care of adults with SCD is largely guided by the 2014 Evidence -Based Management of Sickle Cell Disease: Expert Panel Report. This report used the GRADE method to define and create evidence-based guidelines [7], and informs health care providers’ approaches to screening to prevent diseases or complications of chronic diseases, selecting treatments, monitoring and preventing complications, educating about disease, and counseling for individuals with SCD [8]. Recognizing the importance of disseminating the guidelines and given the ubiquitous access to information technology [9, 10], provider-facing mobile health (mHealth) applications (apps) [11] and telemedicine interventions have been developed to educate providers about SCD guidelines [9, 10, 12]. However, to date no national strategy has been developed to make these SCD-related guidelines; hereafter, referred to as *guidelines*, patient-centered.

We define guidelines to be patient-centered if they are designed to be concordant with the patient’s values and preferences, and would allow them to have an active dialog with their health care providers about their health care [13]. To ensure these patient-centered guidelines are concordant with patient’s values, needs, and preferences, the guidelines needed to be: (1) provided at a health literacy level they can understand; (2) actionable, as using these guidelines effectively requires an active dialog with their health care provider about specific action items in their health care; and (3) a document that they could access and would use as a reference for their medical care. Developing educational material such as patient-centered guidelines can improve disease-specific knowledge [14–20]. Low SCD-specific knowledge is considered a modifiable risk factor associated with substantial negative impact on health outcomes and higher acute health care utilization among individuals with SCD [21–23]. Adapting guidelines to be patient-centered can engage individuals with SCD and their families, thereby having the potential to improve SCD-specific knowledge and decrease health care utilization.

Clinical practice guidelines are written for health care providers, and there is increasing interest in creating a guideline version for patients and their caregivers [24–28]. In SCD, these guidelines have been created for providers but not for patients. In addition, these guidelines are not always actionable for patients. The guidelines do not have action items that patients can follow to help with self-management or preventive measures they can discuss with their provider, thereby limiting their ability to engage in their own care. To improve the quality of care delivered to individuals with SCD and their caregivers, we developed a novel recursive process to create a single set of patient-centered guidelines using community-engaged research in a rare disease, SCD, where guidelines and high-quality evidence have been created for providers. Community-engaged research involves creating a partnership with community members, organizational representatives, health care providers, and researchers where all contributions are equal, shared decision making occurs, and everyone has ownership of the entire research process [29]. Community-engaged research is different than community-based participatory research as community-based participatory research is defined by working with an organization, which serves as a community partner who actively participates in the research. [30–34]. Community-engaged research engages the community to give input on the research questions, but does not necessarily partner with the community in the research process. The aims of this study were to use community-engaged research strategies [35–38] to: (1) explore the research question about if the SCD community wants access to these SCD-related guidelines and how they would want to access them, and (2) to adapt provider-centered guidelines to be patient-centered for the SCD community to improve productive discussions with a prepared, proactive practice team.

## Methods

### The engagement process

**Fig. 1:**
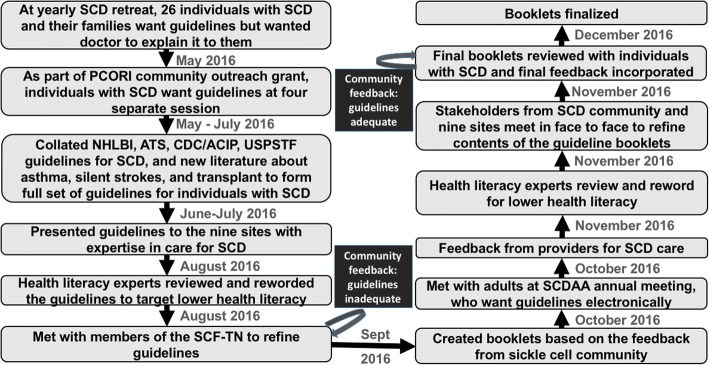
Flow chart and timeline of creation of the guideline booklets for individuals with sickle cell disease (SCD). NHLBI: National Heart, Lung, and Blood Institute; ATS: American Thoracic Society; CDC: Centers for Disease Control; ACIP: Advisory Committee of Immunization Practices

Using community-engaged strategies to include the SCD community through community-engaged research is crucial in developing patient-centered guidelines. These patient-centered guidelines need to be useful to providers as well as to individuals with SCD and their caregivers. Community-based organization partners (Sickle Cell Disease Association of America (SCDAA) and Sickle Cell Foundation of Tennessee (SCFT)) guided the process of community-engaged research and served as a conduit between health care providers, researchers and individuals with SCD. Through our partnership, we implemented several different community-engaged research strategies: (1) three community forums, (2) four community listening sessions, (3) weekly teleconferences, and (4) a two-day in person meeting. The iterative process to adapt the guidelines occurred from May 2016 to December 2016 (Fig. 1). The details of each strategy are discussed in the sections below.

The engagement process started with a needs assessment at the Vanderbilt University’s annual SCD retreat community forum. This needs assessment aimed to discover whether the SCD community (individuals with the disease, parents of children with disease) wanted guidelines. Once the needs assessment was completed, and the SCD community expressed desire for guidelines during this community forum, a series of weekly teleconferences commenced.

The objective of these weekly teleconferences was to include providers and community representatives in an effort to curate guidelines so they would be acceptable to both providers at SCD centers across the United States, as well as individuals with the disease. Guidelines that are acceptable to providers and patients could allow for improved joint decision making.

These weekly teleconferences, attended by community-based organization leadership and experts in the care of SCD from across the country at nine academic institutions, were held over a six-month period. These experts in SCD included hematologists, primary care providers, psychologists, researchers, and nurse case managers. All of these experts provided care for individuals with SCD. The teleconferences allowed community-based organization leadership to give insight from the SCD community perspective with these experts in SCD present. During these teleconferences, experts in SCD and community-based organization leaders reviewed and curated a set of guidelines from (1) the 2014 “Evidence -Based Management of Sickle Cell Disease: Expert Panel Report” guidelines, (2) the American Thoracic Society guidelines about pulmonary hypertension for SCD, (3) the United States Preventative Services Task Force, (4) the Advisory Committee on Immunization Practices, and (5) recent randomized controlled trial evidence about asthma and silent strokes [39–41]. At the conclusion of these meetings, a set of guidelines was agreed upon that health care providers could use in the care for individuals with SCD. Once this set of guidelines was agreed upon by the SCD experts and community-based organization leadership, these guidelines were refined to be understandable for patients through a discussion with the health literacy experts at Vanderbilt University Medical Center. The health literacy experts gave recommendations on language that reduced the reading level to the 5th–7th grade reading level using the Flesch–Kincaid readability tests, and substituted medical jargon with layman’s language.

Now with a curated, more understandable, acceptable set of SCD guidelines, the community-based organization leadership recommended more community involvement to get a patient perspective, and make them useful to the community. This advice prompted implementation of community engagement strategies. These community engagement strategies included community forums and community listening sessions, which served as a continuation of the needs assessment as well as a platform to review guideline content. These strategies allowed for community feedback about the content’s understandability, actionability, and usability. The engagement strategies are described in detail below.

After feedback was obtained, another iteration of the cycle was required, where the SCD experts and community-based organization leadership discussed the revised content, health literacy experts reviewed and modified these updates to make the content understandable at a 5th–7th grade literacy level, and the SCD community provided feedback (Fig. 1). The process concluded with a two-day in-person meeting held at Vanderbilt University Medical Center to allow for face-to-face discussion among all members of the research team (experts in SCD care, leadership from the community-based organizations, and individuals with SCD and their caregivers). During this face-to-face meeting, Guideline booklets were reviewed and feedback was incorporated.

### Recruitment methods for community forums and community listening sessions:

A convenience sampling was used to recruit participants for the community forums and community listening sessions. The effectiveness of these strategies is demonstrated in studies eliciting community input on an area of importance for the community not previously described [42–44]. The researchers elected to implement both community forums and community listening sessions to provide an opportunity for feedback in research-led and community-led environments. The participants for the community forums were attendees of the Vanderbilt University’s annual SCD retreat that took place in central Tennessee, the SCDAA annual meeting held in Baltimore, and the SCFT monthly meeting held in Nashville. Participants were either individuals with SCD or caregivers of individuals with SCD. The participants at the Vanderbilt University’s annual SCD retreat were individuals that are cared for at the Vanderbilt Meharry Sickle Cell Center for Excellence. These individuals were invited to participate in a weekend of camaraderie and an educational session about guidelines. Participants at the annual SCDAA meeting included a group of adolescents and young adults with SCD that were attending the annual meeting. This group was invited to come to a session discussing the guidelines as part of the itinerary of the meeting. Participants from the SCFT community forums were invited to be a part of their monthly meetings that took place in Nashville.

Community listening sessions were facilitated by a trained representative from the SCFT. Participants for the community listening sessions were individuals with SCD and their caregivers (*n* = 43) in two urban and two rural communities throughout three regions (West, Middle, and East) in Tennessee. Participants were recruited via email distribution through the SCFT. Interested participants were then invited to attend a scheduled session held in a community setting. Sessions lasted approximately 1.5 h each. The sessions were audio-recorded and notes were scribed. All sessions were commercially transcribed and used verbatim for analysis.

### Community forums and community listening sessions:

#### Community forums

Community forums were planned for individuals with SCD and their caregivers to explore the research question about desire to access guidelines and then to adapt the guidelines to be patient-centered. Similar to other studies, these forums were led by researcher staff. The forum started with multiple-choice questions to explore our research question about if individuals with SCD and their caregivers wanted access to the SCD-related guidelines, and then how they would want to access these guidelines. Other questions included respondent demographics and perceptions of impact of guideline usage on personal health. Individuals with SCD, their caregivers, or both used response clickers, remote control-like devices, to submit anonymous responses to straw poll surveys about information technology and guidelines. The responses to the multiple-choice answers were evaluated with quantitative methods described below.

After the multiple-choice questions were completed, these meetings focused on guideline content and what changes should be made to make them patient-driven. Qualitative feedback was recorded by participants and the research team on guideline booklets and collected by the research team at the end of the session. Respondents voluntarily attended the community forums and contributed data through participation in the audience response activity.

#### Community listening sessions

Community listening sessions (*n* = 4) are a qualitative method of obtaining feedback that allows individuals to express their views on an issue in a more wide-ranging manner [45]. These sessions were different from the community forums as they were led by trained representatives from the SCFT and not researchers, and allowed qualitative feedback about a range of issues (e.g., disease management, nutrition, and provider interaction) including patient-centered guidelines.

### Data analysis for community forums

For quantitative data, descriptive statistical methods and tests were used to summarize demographics and responses to community forum multiple-choice questions. Continuous variables were summarized and tabulated in terms of totals and percentages. Fisher’s exact tests were performed to determine differences in: (1) the demographics of the different community forums, (2) question responses among the different community forums, and (3) channels that respondents wanted the guidelines to be communicated. Statistical tests were performed using R version 3.2.2 [46]. *P*-values of < 0.05 were considered significant.

For qualitative data, the participants were asked about the importance of each point brought up and if more than one person agreed in the importance of the point, the point was recorded by the research team. After conclusion of the community forums, all points recorded were reviewed by the research team and the team conducted a thematic analysis from the data obtained and explored common themes across group responses using grounded theory [47, 48].

### Qualitative data analysis for community listening sessions

An inductive, qualitative content analysis approach was used to analyze the data from the community listening sessions. Members of the research team trained in qualitative data analysis reviewed these data and conducted a thematic analysis. Common themes across participant responses were explored using grounded theory [47, 48].

### Ethical aspects

Participants were informed that their responses would be used for quality improvement purposes, such as the development and implementation of an information technology system for communicating guideline information. Informed consent was waived for this IRB-exempt, HIPAA-compliant, retrospective review of prospectively acquired quality improvement data. The Vanderbilt University Medical Center IRB approved this work.

## Results

### Individuals with SCD and their caregivers used technology, wanted to know about guidelines and were interested in having the guideline educational material delivered in different ways

A total of 64 individuals with SCD and their caregivers were included in the community forums. There were more adults and caregivers who participated in the sessions than children and adolescents (Table 1). The majority of participants at the SCD retreat were caregivers, and at the SCDAA and SCFT were adults. The groups had significantly different demographics (*p* < 0.01).

**Table 1:** Demographics of SCD and caregivers who participated in the community sessions. Fisher’s exact test was used to determine the *p*-value of differences between the different forums and age groups

	Age less than 15	Age = 15–25	Age older than 25	Caregivers	*p*-value
Sickle Cell Retreat	0	0	1	26	< 0.01
Sickle Cell Disease Association of America	2	9	15	0	
Sickle Cell Foundation of Tennessee	1	1	9	0	

In the first community forum (SCD retreat, *n* = 27), 25 of 27 participants (93%) wanted to know what the guidelines were, providing strong motivation to proceed with the effort to adapt the SCD guidelines. Among the 64 participants pooled from all four venues, over half (58%) knew what a medical guideline was, and 91% would want to know what the content of the guidelines are (Table 2). A majority of individuals with SCD and their caregivers wanted the guidelines to be available in multiple formats, but only 44% want them on a patient portal (p < 0.01) (Table 2). The responses to questions were significantly different for using apps for the health of someone with SCD (*p* = 0.01), wanting the guidelines on the patient portal (p < 0.01), and wanting the guidelines in a mobile app (*p* = 0.03).

**Table 2:** Responses to questions asked at the different community forums. An ‘x’ means the question was not asked in that forum. Fisher’s exact test was used to calculate the p-value

Question	Response	SCD retreat (n = 27)	SCDAA (*n* = 26)	SCFT (*n* = 11)	*p*-value
Do you have a smart phone like a Samsung, iPhone, Android?	Yes	25	24	10	0.39
	No	2	0	1	
Do you use your phone for text messaging?	Yes	27	23	10	0.56
	No	0	1	0	
Do you use email for the health of someone with SCD?	Yes	x	14	8	0.43
	No	x	10	2	
Do you use social media like Facebook, Twitter or Instagram for the health of someone with SCD?	Yes	x	15	5	0.25
	No	x	6	6	
Do you use mobile apps for your health?	Yes	13	10	7	0.42
	No	14	15	4	
Do you use apps for the health of someone with SCD?	Yes	8	15	7	0.01
	No	19	6	4	
Do you know what a medical guideline is?	Yes	x	11	8	0.24
	No	x	10	2	
Would you want to know what these guidelines are?	Yes	25	18	10	1
	No	2	1	0	
Would you feel you could provide better care to the family member with sickle cell if you knew what the guidelines were?	Yes	20	19	9	0.14
	No	7	1	1	
Would you want your doctor or nurse explain the content of the guidelines to you?	Yes	25	17	10	1
	No	2	2	1	
Would you want a paper copy of the guidelines?	Yes	20	16	8	1
	No	7	5	3	
Would you want to receive text messages about the guidelines (short ones daily)?	Yes	22	15	7	0.77
	No	4	2	2	
Would you want the guidelines available on your patient portal?	Yes	x	6	9	< 0.01
	No	x	18	1	
Would you want guidelines in Facebook/Instagram/Twitter?	Yes	25	17	7	0.1
	No	2	7	2	
Would you want guidelines in mobile app?	Yes	17	13	10	0.03
	No	8	11	0	
Would you want guidelines in app over Facebook?	Yes	x	19	9	1
	No	x	3	1	
Facebook to communicate about guidelines and other things	Yes	25	x	x	x
	No	2	x	x	

Individuals with SCD used technologies at different rates to find health information or to track their health. A large majority of participants used their smartphones and cell phones for texting (95 and 98%, respectively). More than half used email and social media for their health (65 and 61% respectively), but less than half used smartphone health apps (47%). The apps that were most commonly used included various patient portal apps and the VOICE crisis alert app [49].

In the community listening sessions (*n* = 4), the major themes from respondents (*n* = 43) included: (1) a desire for guidelines and educational material from physicians on how to manage their disease; (2) a need for information on how to access the guidelines; and (3) a desire to learn more about how to use the guidelines to communicate with health care providers. Respondents, who had accessed the existing guidelines, also expressed that the educational material is written at a level that they cannot understand.

### Guideline content was modified by input from many different members of the SCD community, including individuals with SCD and their caregivers

Adapting the SCD guidelines was an iterative process. Health literacy experts from the Vanderbilt Effective Health Communication Core edited the original guidelines based on grade-level metrics. The edited guidelines were brought back to the SCD community for comment. The main themes that developed from the SCD community included: (1) creating more explanations for medical concepts; (2) streamlining the format and organization of the information; (3) identifying which content was actionable; and (4) using more visual representations of the content. They recommended that the guidelines could be “made more actionable” and described the initial version as “too wordy”. Some examples of specific changes from these themes are presented in Table 3. The community members also had additional ideas for future sections of the guidelines booklet including: (1) a section on aging with SCD; (2) a section on overall healthy living, including diet, exercise, and other general concepts about how to live a healthy life with SCD; and (3) a section on preventing complications including issues like high altitudes, cold, heat, dehydration, and other anticipatory care.

**Table 3:** Themes and examples of specific feedback about initial content of guidelines

Themes	Examples of feedback
Creating more explanations for medical concepts	“There was still a bit of medical jargon in there that either needs to be removed, or there should be a definitions and examples page”
	“For drugs, [it] would be nice to have definition, examples, side effects ([especially] Hydroxyurea)”
	“I would want symptoms, or what do we mean by ‘gallstones’ or ‘discomfort’”
	“Also, [we] want more about why SCD causes this or affects this, that these complications happen over years”
Streamlining the format and organization of the information	“Categories need to be better laid out, for example, by age would be better”
	“More small sentences and bullet points, not long paragraphs”
	“Remove all the ‘if you are XX age’ … and make content only appear in certain sections by age”
	“There is too much information that may not be relevant to a person at their [current] age, and they would just want relevant information [filter by their age]”
	“Some things were repetitive like vaso-occlusive episode, and there should be a definition for the section, but then just call the episode a ‘pain crisis’”
	“Combine depression screening, or at least make [the recommendations] by age, and again, less wording, more bullet points”
Identifying which content was actionable	“Make the verbiage more actionable, for example, if you don’t have a pain action plan – get a pain action plan”
	“I can’t tell what is actionable [in these guidelines]”
Using more visual representations of the content	“I would like a vaccine schedule in the content and more pictures”
	“We need more pictures”

Feedback was incorporated and another version of the guidelines booklet was developed. After this iterative process was completed two times, only minor changes were recommended, which resulted in the final version of the guidelines booklet. Some examples of changes made because of feedback from the SCD community included: (1) creating actionable checkboxes for items that individuals with SCD and their caregivers can take action, (2) having examples of forms such as pain action plans, (3) including more bullet points with shorter sentences, (4) explaining more medical concepts such as pulmonary hypertension, and (5) organizing booklets into the following major sections: Staying Well; Treating Sickle Cell Disease, Managing Sickle Cell Complications, and Other Conditions that can Affect Sickle Cell Disease (Table 4). At the end of the feedback, 100% of participants said the guidelines were understandable, 88% said the guidelines were actionable, 90% would use these guidelines to keep track of their SCD, and 100% would use these guidelines to discuss their medical care with their providers. Consistent with feedback from participants, the future goal will be to provide widespread access to this version of the guidelines via a paper-based version and via incorporation into an mHealth app to the SCD community and other SCD centers [50, 51].

**Table 4:** Examples of strong SCD NHLBI recommendation and evidence for individuals with SCD according to the NHLBI guidelines and recent randomized controlled trials. Boxes denote action items an individual can take based on the NHLBI guidelines.

Provider Guidelines	First version of Patient-Centered Guidelines	Patient-Centered Guidelines after iterative process described in Fig. 1
Use an individualized prescribing and monitoring protocol (written by the patient’s SCD clinician) or an SCD-specific protocol whenever possible to promote rapid, effective, and safe analgesic management and resolution of the vaso-occlusive crisis in children and adults (Pain action plan)	Sometimes blocked blood vessels can cause a sickle crisis, which involves severe pain. In order to help you quickly and safely during these crises, talk to your SCD doctor about creating a set of rules specific to your needs. Include rules about getting medication, and how often your doctor will check in with you.	What can be done at home to manage the pain? • A pain action plan describes how to manage sickle cell pain at home. Action plans should be used as soon as the pain starts. An example of a pain action plan can be found in *Forms*. □ Ask your health care provider about creating a written pain action plan that works for you. • Call your health care provider if the pain does not get better, or gets worse even though you are using your pain action plan.
Treat avascular necrosis with analgesics and consult physical therapy and orthopedic departments for assessment and follow-up	If you experience discomfort caused by your bones not getting enough blood supply, sometimes called avascular necrosis, talk to your doctor about taking pain medications. Your doctor may recommend you see physical therapy and orthopedic doctors.	What is the treatment for avascular necrosis? • Treatment options depend on how much the joint is affected. □ Talk with your health care provider about sending you for physical therapy to make the muscles around the joint stronger and more flexible. □ Talk with your health care provider about ways other than medication to manage your pain: • Using heat, such as a warm compress, warm bath, or a heating pad • Gently massaging the area that hurts • Doing something to distract you from the pain like listening to music, drawing, watching TV, or writing in a journal • Doing deep breathing and relaxation exercises □ If approaches without medications do not help, talk with your health care provider about medications to control the pain. □ A special health care provider (orthopedic surgeon) may see you for additional evaluation and treatment. ◦ Sometimes surgery is needed if other treatments do not work.
In infants 9 months of age or older, in children, in adolescents, and in adults with SCA, offer treatment with hydroxyurea regardless of clinical severity to reduce complications (e.g., pain, dactylitis, ACS, anemia) related to SCD	If your child with SCD is between 9 months old and 18 years old, check with his or her doctor about using the drug hydroxyurea to try to lessen complications of SCD. Examples of complications include pain, finger/toe swelling and redness, and low red blood cell count.	What is Hydroxyurea? • Hydroxyurea is a medication that increases the amount of fetal hemoglobin in red blood cells. Fetal hemoglobin helps to keep the red blood cells from sickling. • Hydroxyurea is not a cure for sickle cell disease, but it may help *decrease* many of the complications of the disease, including: ◦ Anemia ◦ Pain episodes ◦ Episodes of acute chest syndrome ◦ The need for blood transfusions ◦ Long hospital stays • In adults, some studies have found that hydroxyurea helps you live longer. Who should take hydroxyurea? • Talk with your health care provider about hydroxyurea if: □ You have sickle cell anemia (type SS or sickle beta thalassemia zero) □ You have sickle cell disease (type SC or sickle beta thalassemia plus) and have pain or other sickle cell complications that affect your ability to do your daily activities or that affects your quality of life.
No definition in guidelines	Not included in the first version	What is pulmonary hypertension? • Pulmonary hypertension is more common in people with sickle cell disease than in people without sickle cell disease. • Your heart is made up of two pump systems. The right side of your heart pumps blood to your lungs to pick up oxygen. The left side of your heart pumps blood to the rest of your body. • Blood pressure measured with a cuff on your arm is measuring the pressure it takes to pump blood to your body. High blood pressure is called hypertension. • The pressure it takes to pump blood to your lungs can also be measured. High blood pressure here is called pulmonary hypertension.

## Discussion

As evidence -based medical guidelines become standard for practicing medicine [52] and are applied as metrics for quality of care [53], individuals affected by the disease should be partners in adapting and implementing such guidelines. To our knowledge this is the first application of community-engaged research principles in SCD to modify established evidence -based guidelines and current best evidence to make them more directly useful for individuals with SCD, and their caregivers to use with health care providers. Over a six-month period, the process weighed input from all stakeholders equally (individuals with SCD their caregivers, and SCD experts involving SCD health care providers) including SCD community-based organizations. The process started with understanding if the SCD community members (individuals with the disease, parents of children with disease, and leaders of community-based organizations for SCD) wanted usable guidelines and how the SCD community wanted access to the guidelines. In an iterative process that included a multidisciplinary team of SCD experts, health literacy experts, and the SCD community, published evidence -based SCD guidelines were transformed into a version that is patient-centered. To be patient-centered, the guidelines were adapted to be understandable, actionable, accessible, and useful for individuals with SCD and their caregivers. This patient-centered guideline booklet was agreed upon by SCD health care providers from across the United States and rated as widely acceptable by SCD community members, making it a feasible tool for use by health care providers and individuals with SCD and caregivers in making shared decisions about their care. The booklet also has the potential to engage and activate patients and improve their *SCD-specific knowledge*. Engaged and activated patients have better health outcomes including better diabetes control, less depression, more preventive cancer screening tests for women (Pap smear and mammography); and lower costly utilization (emergency department visit or hospitalization) [54, 55]. Improving *SCD-specific knowledge* can lower annual rates of emergency department utilization and hospitalizations in individuals with SCD [21].

Limited literature exists to describe a process of adapting and “translating” clinical practice guidelines to become more patient-centered. Knowledge of clinical practice guidelines among patients is low and there is a growing desire for patients and their care providers to embrace and have the knowledge of these guidelines [56–58], similar to our findings in SCD. Engaging patients in clinical guideline development and review has also been described [25, 59–61]. Some of these studies described the following potential strategies that mirror our community-engaged research approach: including patient input, having appropriate stakeholders at the table (e.g., consumer stakeholders), and convening multiple meetings with sufficient time. By utilizing these strategies, we were able to mitigate barriers previously described to create patient-centered guidelines including: difficulty understanding the discussion or content, resisting patient input by healthcare providers, allowing a dialog among patients, caregivers, community-based organizations, and health care providers, and having sufficient time for discussion.

Few examples of patient-centered guidelines exist in the literature today, and most of those examples are from the United Kingdom and Europe [61–66]. Description of how guidelines were adapted for patients was limited to a few studies, all outside of the United States [62–64]. These studies, like ours, used experts and patients to modify the wording of guidelines to be more patient-centered, while maintaining original meaning [62, 63]. Our study included community-based organizations in addition to patients, and used an iterative process to arrive at a final version, where these prior studies did not use community-based organizations in the process and finalized their patient-centered guidelines during or after only a single meeting of all stakeholders. While most studies used lay language to explain complex medical terminology, Kiltz et al. described keeping some original scientific language, as modifying them did not improve understanding of the concept. Our finalized booklets also kept some scientific language, such as “pulmonary hypertension”, “hydroxyurea”, and “sickle cell beta thalassemia”, for the same reasons. Similar to our study, other studies mentioned a list of ideas that patients desired to be included in the next update of the guidelines. Another article by the Scottish Intercollegiate Guidelines Network (SIGN) group demonstrated that the value and usefulness of the patient-centered guideline was based on how it informed the public, linked information to actions, and empowered people in interacting with their healthcare providers [67]. Our findings were similar, with the desire for actionable content and information that was clear and understandable. We also found that individuals with SCD had a desire to know how to stay well when having their disease, and wanted a wellness section. The only manuscript about patient-centered U.S. guidelines was McClure’s adaptation of clinical practice guidelines for people with cancer [66]. These guidelines were adapted from the National Comprehensive Cancer Network (NCCN) clinical practice guidelines that provide diagnosis, evaluation, and treatment options about people’s cancer [66, 68]. Our work does not explain the methods of adaptation or if and how people and communities with cancer were engaged in the adaptation of the guidelines. Our study expanded upon previous literature by describing the methods of how to create patient-centered guidelines using community-engaged research for U.S. guidelines on SCD, and illuminated the importance of input from the SCD community into the adaptation of existing clinical guidelines to be most understandable, actionable, accessible, and useful for them.

In the initial three meetings with the community, a majority of individuals with SCD and their caregivers could not define a medical guideline, but almost all participants wanted the guidelines to be available to them once they understood what a guideline was. The SCD community wanted the guidelines available in multiple formats, with an overwhelming majority wanting them to be explained by a health care provider, and about 70% wanting them in paper and electronic formats. Patient portals were distinctly less appealing, with less than half wanting to access the guidelines in this way.

In our prior work, we demonstrated that individuals with SCD and their caregivers want to access technology when asked: “How would you prefer to be contacted to learn about potential research studies?” [69] The results of the community-engaged research project confirm that technology is emerging as a preferred medium for individuals with SCD and their caregivers to learn about their care, specifically evidence -based guidelines. However, individuals with SCD and their caregivers are unsure of the optimal technology for tracking and managing their health. This health care technology gap in the SCD community will make Meaningful Use governmental regulations that focus on the use of health technologies to promote improved outcomes in patients, difficult to implement [70]. As a result of the government mandate, many health care systems and providers are using patient portals to meet these Meaningful Use regulations [71], which is in contrast to what individuals with SCD and their caregivers wanted to access guidelines. Further work is required to further describe and develop ways to address this health care technology gap.

Community involvement in translating the evidence-based guidelines from documents intended for health care providers into patient-accessible content was of paramount importance. After the family retreat, the participants demonstrated their desire to have access to guidelines. The process started with the expertise of SCD providers across the country to curate the guidelines into the essential knowledge content that health care providers would use to care for individuals with SCD. As previous literature has demonstrated, educational material for SCD is often developed at too high of a reading level [72]. Therefore, our next step was to work with a health literacy expert team to make the language more accessible; however, when we approached the community with the adapted guidelines, the community could not extract meaningful content and did not yet find the guidelines useful. This community input led to a set of improvements including changing the format of the guidelines from dense paragraphs translated from the original medical documents into a digestible set of explanatory points, and adding action items that could easily be used by the SCD community. The iterative process allowed all groups to converge on a guideline booklet that was factual, comprehensive, patient-centered, actionable, and most importantly, one that individuals with SCD and their caregivers would want to use in their discussions with health care providers.

There were several limitations to our community-engaged research approach. Our data collection strategy did not allow for subgroup analysis (i.e., how many people selected each multiple-choice option) across the population. However, we do not believe this limitation significantly impacted our results as for most study findings and guideline adaptations (i.e. should we have guidelines, should the guidelines have a paper format) there were no substantial alternate findings or opinions. Further, we elected to use a clicker system to increase the likelihood of honest feedback from the self-selected groups that participated in the community forums. We realize that the health care providers participating in this project may not be representative of SCD health care providers across the country. To limit biases, we deliberately selected published evidence-based guidelines or new published evidence to determine what evidence -based guidelines should be included and presented to caregivers. Specific topics that were included in our booklet but not the NHLBI guidelines were depression screening, role of hematopoietic stem cell transplant for cure, screening for silent strokes, and screening for asthma. While we obtained feedback from the national SCDAA meeting where individuals with SCD from around the United States meet, our results may not generalize to the broader population of individuals living with SCD or their caregivers. Finally, we have not evaluated uptake of using the guidelines by patients and providers; however, this is an active area of research for our group and 100% of individuals with SCD and caregivers state they would like to use these guidelines.

The emphasis of providing the guidelines for care in SCD has been directed toward health care providers. SCD experts have developed an mHealth apps for health care providers to increase the adoption of the 2014 guidelines [11]. Others have created educational programs using telemedicine to educate providers about these guidelines [12]. However, to date no national strategy has been developed to make these guidelines patient-centered, accessible and comprehensible for individuals with SCD.

We undertook this activity as an extension of Vanderbilt’s Sickle Cell Center of Excellence approach of providing individuals with SCD and their caregivers with evidence -based knowledge and action items to improve their overall care and satisfaction with the care [73]. This work describes a community-engaged process that will aid in the adaptation of provider-centered guidelines for individuals and caregivers of a hematologic disease to become informed and activated. This patient-driven approach resulted in a guideline booklet that the SCD community found useful and would want to use for productive discussions with a prepared, proactive practice team [74]. As guidelines continue to evolve, this patient-driven process will be useful for adapting future evidence-based care recommendations to be patient-centered, comprehensible, and accessible. Next steps include evaluating these guidelines and their ability to activate individuals with SCD, improve *SCD-specific knowledge*, and decrease acute health care utilization.

## Conclusions

In this study, we engaged the community using community-engaged research strategies and found that individuals with SCD and their caregivers wanted access to the same SCD guidelines relied on by health care providers, and wanted these guidelines through multiple channels, including technology. Based on these positive results, we used community-engaged research involving health care providers, community-based organizations, and individuals with SCD, to adapt guideline material for the SCD community. The adapted patient-centered guidelines have the potential to improve the SCD community’s knowledge of their SCD, and serve as a conduit for productive discussions with a prepared, proactive practice team. The approach presented in this manuscript is potentially generalizable to adapting guidelines in other diseases designed for providers, to be patient-centered.

## Additional files

Additional file 1: Raw data. (XLSX 8 kb)

Additional file 2: List of questions asked at the community forums. These are the questions that were asked of participants at the community forums. (XLSX 12 kb)

## Abbreviations

SCD: Sickle cell disease
SCDAA: Sickle Cell Disease Association of America
SCFT: Sickle Cell Foundation of Tennessee
